# 

**Published:** 1886-11

**Authors:** 


CONTENTS:

ARTICLE I—Anæsthesia vs. Asphyxia. By Dr. S. J. Hayes............... 289
" II—Southern Dental Association........................................ 296
" III—Oxygen as a Therapeutic Agent. By C. E. Ehinger, M. D. 327

EDITORIAL, ETC.

Dental Students........................................................ 331

The Point of Death........................................................ 332

BIBLIOGRAPHICAL.

A Practical Treatise on Mechanical Dentistry. By Joseph Richardson, M. D., D. D. S........................................ 334
Index to the Periodical Literature of Dental Science and Art. By J. Taft, M. D., D. D. S................................ 335

MONTHLY SUMMARY.

Improved Method of Operating for Cleft Palate.......................... 335
Swallowing a Set of Artificial Teeth.................................... 336

